# Feasibility and validity of ecological momentary cognitive testing among older adults with mild cognitive impairment

**DOI:** 10.3389/fdgth.2022.946685

**Published:** 2022-08-05

**Authors:** Raeanne C. Moore, Robert A. Ackerman, Madisen T. Russell, Laura M. Campbell, Colin A. Depp, Philip D. Harvey, Amy E. Pinkham

**Affiliations:** ^1^ Department of Psychiatry, University of California San Diego, La Jolla, CA, United States; ^2^ Department of Psychology, School of Behavioral and Brain Sciences, The University of Texas at Dallas, Richardson, TX, United States; ^3^ San Diego State University/University of California San Diego Joint Doctoral Program in Clinical Psychology, San Diego, CA, United States; ^4^ Veterans Affairs San Diego Healthcare System, San Diego, CA, United States; ^5^ Psychiatry and Behavioral Sciences, University of Miami Miller School of Medicine, Miami, FL; ^6^ Research Service, Bruce W. Carter VA Medical Center, Miami, FL, United States

**Keywords:** ecological momentary assessment, ambulatory assessment, smartphones, Alzheimer's disease, adherence, psychometrics

## Abstract

It is critical to intervene early in the mild cognitive impairment (MCI) stage of the Alzheimer's disease trajectory, but traditional cognitive testing methods are costly, burdensome, and difficult to access. We examined adherence and validity data to a 30-day self-administered ecological momentary cognitive testing protocol among a sample of older adults with MCI and cognitively normal controls to evaluate feasibility, tolerability, and initial validity in comparison to standard neuropsychological tests. Participants included 48 participants with MCI (Mean age = 72 years, SD = 7 years) and 46 demographically-matched cognitively normal (NC) control participants (Mean age = 70 years, SD = 7 years). Participants completed traditional neuropsychological testing to determine MCI status, followed by 30 days of remote ecological momentary cognitive testing. Ecological momentary assessment (EMA) surveys were administered 3 times per day for 30 days (possible total = 90), and mobile cognitive tests were administered every other day (for a total of 15 administrations). Mobile cognitive tests included the Variable Difficulty List Memory Test (VLMT; measure of learning and memory), Memory Matrix (measure of visual working memory), and the Color Trick Test (measure of executive function). EMA and mobile cognitive test adherence, fatigue effects, mobile cognitive test performance and group differences, and psychometrics (reliability, convergent validity, ceiling effects, and practice effects) were examined. Overall mean-level adherence to the mobile cognitive tests was 85% and did not differ by MCI status. The reliability of stable between-person individual differences for the VLMT and Memory Matrix were very high. Moreover, although the reliability of within-person change for Memory Matrix was adequate, the corresponding reliability for VLMT was somewhat low. Averaged performance on the mobile cognitive tests was correlated with lab-based tests measuring the same construct. Participants with MCI performed worse than NCs on the VLMT and Color Trick Test, and there was no evidence of fatigue effects for these two tests. These findings support the feasibility and potential for ecological momentary cognitive testing to support clinical trials and for measuring cognitive changes over time in persons with increased risk for Alzheimer's disease such as those with MCI.

## 1 Introduction

Research that examines cognitive functioning has traditionally taken place in a lab with paper and pencil neuropsychological testing; however, there are barriers with this method, including high cost, time burden, and access to testing locations which are limited by transportation and uneven distribution in rural or remote areas. As a result, neurocognitive testing is infrequently repeated, if at all. Ecological momentary cognitive tests (EMCTs), which are brief and repeatable cognitive assessments that are self-administered *via* smartphone in participants' own environments, may be a valuable complement to traditional neuropsychological testing that can help overcome some of these barriers (1–4).

There are several advantages to EMCTs that may make them well suited for use in clinical trials. Cognition can fluctuate from day to day, which makes it is difficult to determine what should be considered a real change on neuropsychological testing from one time point to another. This is particularly problematic when trying to examine improvement over time (e.g., recovery from stroke) or cognitive decline as seen in Alzheimer's disease and related dementias. Alzheimer's disease is the most common cause of dementia in older adults (5) and places significant financial and emotional burden on affected families, not to mention the financial impact on healthcare systems. Therefore, it is no surprise that there are currently hundreds of ongoing clinical trials aimed at prevention of and intervention in Alzheimer's disease and related dementias (6).

To date, pharmacological interventions have been slow to show reductions in cognitive decline, and no treatments have been able to reverse cognitive decline despite some evidence for slowing disease progression; however, many of these studies use less-than-optimal cognitive outcome measures. For example, the Alzheimer's Disease Assessment Scale–Cognitive Subscale (ADAS-Cog) has been shown to have significant ceiling effects in those with normal cognition and mild cognitive impairment (MCI) and there are concerns about its ability to detect cognitive changes early in the disease course (7–9). Given that EMCTs can be given over multiple days, EMCTs may be a cost effective and time efficient method to establish a more accurate baseline for cognitive functioning and to detect person-specific changes more sensitively over time. Such procedures could also allow for dynamic titration of difficulty in order to more effectively probe variation in performance.

EMCTs can also be paired with other technologies such as ecological momentary assessment (EMA) or wearable devices (e.g., actigraphy to objectively assess physical activity and sleep). Therefore, observational studies or interventional studies can examine how mood, activities, sleep, and other fluctuating daily-life factors associate with cognition over time without relying on retrospective recall, which is particularly relevant to persons with memory impairments (e.g., 10, 11). Utilizing EMCTs to examine cognition in a person's everyday life with different contextual variables could lead to person-specific intervention strategies (4).

Additionally, the use of EMCT may reduce the number of in-person visits, which could reduce the burdens of time and transportation, particularly for participants that live in rural areas and older adults with mobility limitations. The tradeoff is that technology familiarity may impact one's ability to engage in EMCTs and is something to be mindful of in this group. However, a study conducted in 2021 by the Pew Research Center found that 83% of those aged 50–64 own a smartphone and 61% of adults aged 65 + own a smartphone, indicating that the majority of older adults are already engaged with smartphone technology (12). To date, there have been a handful of studies by other groups utilizing smartphone-based mobile cognitive testing among cognitively normal older adults (e.g., 10, 13, 14) and older adults with MCI (e.g., 15, 16), all of which have demonstrated feasibility, good adherence, and promising initial psychometric properties for use of these tests in this population.

Despite the clear appeal of EMCT in aging research, there are some current limitations. For example, a recent systematic search and evaluation found that the majority of currently-available commercial-grade app-based tools to assess cognition lack validity data for their assessments (17). This is concerning, as an absence of validity data in these tools could lead to unreliable information about possible cognitive impairment. Therefore, we present adherence and validity data in a group of older adults with and without MCI for three NeuroUX EMCTs assessing the domains of memory and executive functioning: 1) Variable Difficulty List Memory Test (VLMT), which is a verbal list-learning test in which we administered 6-word, 12-word, and 18-word versions; 2) Memory Matrix, a visual working memory task; and 3) Color Trick Test, an executive functioning task examining inhibition using a Stroop-Type paradigm. The aims of the study were to examine the 1) adherence to the 30-day EMCT protocol, 2) fatigue effects, 3) EMCT task performance and group differences, and 4) EMCT psychometrics, including reliability, convergent validity (compared to traditional neuropsychological tests), ceiling effects, and practice effects.

## 2 Materials and methods

### 2.1 Participants

Participants were English-proficient individuals aged 50 or older who met criteria for any subtype of mild cognitive impairment (MCI) using Jak/Bondi criteria, which require performance of one standard deviation below normative expectations on two different assessments within a single cognitive domain (i.e., memory, attention, language, executive functioning), or cognitively normal (NC) control participants. Exclusion criteria included: (1) presence or history of medical or neurological disorders that may affect brain function (e.g., stroke, epilepsy, Parkinson's disease), (2) presence of dementia, (3) history of unconsciousness for a period greater than 15 min, (4) significant impairment of vision (e.g., blindness, glaucoma, vision uncorrectable to 20/40, color blindness) or hearing (e.g., hearing loss) that would interfere with their ability to complete the study protocol, (5) presence of intellectual disability (defined as IQ < 70), (6) current diagnosis of substance use disorder, (7) or presence or history of a psychotic disorder or bipolar disorder.

Data were collected across three sites between December 2020 and December 2021: The University of Texas at Dallas (UTD), University of California San Diego (UCSD), and University of Miami Miller School of Medicine (UM), resulting in a total of 94 participants (48 MCI, 46 NC). UTD participants were recruited from community advertisements and previous participation in aging-related research studies at the Center for Vital Longevity at UTD. UCSD participants were recruited from word of mouth and posting in the Stein Institute for Successful Aging monthly newsletter. UM participants were recruited from the clinical programs at the Miller School of Medicine Memory Disorders Center, the Florida ADRC, and through advertisements and previous study participants.

### 2.2 Procedures

The study was approved by each University's respective Institutional Review Board, and all participants provided written informed consent. After a brief phone screen, participants completed a baseline visit either remotely *via* Microsoft Teams or Zoom or in-person. During the baseline visit, participants completed a neuropsychological battery. Research staff held a bachelor's degree or higher, and were trained over the course of several weeks, within and across sites, to administer and score the neuropsychological tests accurately. Jak/Bondi diagnostic criteria for MCI were applied to the neuropsychological test data to determine MCI status. The Jak/Bondi diagnostic criteria show a good balance of sensitivity, specificity, and reliability compared to other conventional MCI criteria (18). Study eligibility, all neuropsychological test scores, and diagnoses were reviewed by the first author (RCM). Once eligibility and group status were confirmed, staff contacted participants to set up their smartphones for the EMCT period. Participants could either complete the EMCTs using their personal smartphone or, if they requested or did not own a smartphone, they were provided with a study-owned Android smartphone. Those using study-provided smartphones were trained to operate the device and given a user manual to reduce technological issues. Participants were trained on the EMCT protocol and completed a mock EMA survey and mobile cognitive testing session to allow for technical questions and troubleshooting.

**Figure 1:**
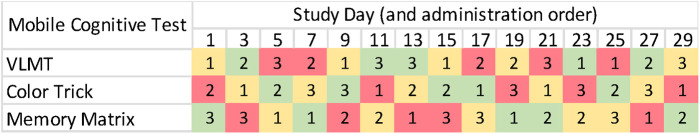
Protocol of mobile cognitive testing administration. *Note.* Difficulty levels are depicted as green (easy), yellow (medium), and red (hard).

For the following 30 days, participants completed the EMCT protocol using the NeuroUX platform (19). Participants were sent text message notifications to take the EMA surveys three times per day. Every other day, participants were asked to complete the three different mobile cognitive tests (i.e., Variable Difficulty List Memory Test, Memory Matrix, Color Trick Task) of varied difficulty along with each of their EMA surveys. The mobile cognitive tests were counterbalanced throughout the EMA period by test type and difficulty level, resulting in a total of 5 easy, 5 medium, and 5 hard conditions of each of the three mobile cognitive tests (see Figure 1). To encourage EMA adherence and help troubleshoot any difficulties, researchers contacted participants if they missed more than three surveys in a row. Participants were compensated up to $190 total for completing the baseline visit ($50) and EMCT sessions (EMA questions only – $0.88; EMA + mobile cognitive tests – $2.25).

#### 2.2.1 Remote visit task modifications

Due to evolving restrictions on in-person data collection during the height of the COVID-19 pandemic, some individuals participated in-person (*n* = 28) whereas others participated *via* remote visits (*n* = 66). For remote appointments, all tasks were completed *via* video conferencing using Microsoft Teams or Zoom meetings and required minimal modification. Participants were asked to complete the visit in a quiet environment away from distractions (e.g., away from other individuals, powering off/silencing unrelated devices) and a screening measure was completed to ensure participants could hear the researcher well and see the PowerPoint materials on their desktop, laptop, or iPad. Researchers also asked participants to refrain from utilizing any performance aids, such as writing down stimulus items, searching for answers on the internet, or seeking help from other individuals.

Tasks that were typically administered orally (Hopkins Verbal Learning Test – Revised (HVLT-R), Number Span Test: Forward) were implemented as is. Tasks that required visual presentations (Wide Range Achievement Test-4 (WRAT-4), Delis-Kaplan Executive Function System Color-Word Interference Test (D-KEFS), Brief Visuospatial Memory Test - Revised (BVMT-R)) were administered *via* video call using a PowerPoint screenshare function. Prior to the baseline visit, research staff instructed participants to prepare four blank pieces of printer paper for the BVMT-R task. Additionally, during the BVMT-R task, after the participant completed each trial drawing, the researcher asked the participant to hold the paper in front of the camera so that a photo could be taken, then instructed them to flip the paper over and place it out-of-sight before beginning the next trial.

### 2.3 Measures

#### 2.3.1 Traditional Neuropsychological measures (lab or remote administered at baseline)

To determine premorbid IQ, the Wide Range of Achievement Test 4 (WRAT-4; 20) word reading subtest was used. The Montreal Cognitive Assessment-BLIND version 7.1 (MoCA-BLIND; 21) was administered to screen for the presence of dementia using established cutoff scores. This version of the MoCA was used for participants who completed virtual visits as well as participants who completed in-person visits. To determine MCI eligibility, the following tests were administered: Hopkins Verbal Learning Test – Revised (HVLT-R; 22), Brief Visuospatial Memory Test – Revised (BVMT-R; 23), Oral Trail Making Test- A and B (24), Digit Span Forward (25), Verbal Fluency – Letter and Animals (25), Multilingual Naming Test (MINT; 26), Number Span Test: Forward (25), and the D-KEFS-Color Word Interference Test (27).

For validity analyses in the current study, we used non-demographically adjusted scores from the HVLT-R (verbal memory), BVMT-R (visual memory), Letter-Number Span (attention/working memory), and D-KEFS Color-Word Interference Test (executive function).

#### 2.3.2 EMA surveys

Each EMA survey asks participants questions about their daily functioning, including where they are (dichotomized as “at home” versus “away”) and who they are with (dichotomized as “alone” versus “with others”). The EMA surveys also generally queried participants' mood, cognitive concerns, substance use, pain, and sleep as additional questions but data are not reported here.

#### 2.3.3 Mobile cognitive tests

See Table 1 for a list of the mobile cognitive tests, the cognitive domains assessed, completion times, and screenshots.

**Table 1:** Mobile cognitive tests.

Mobile Cognitive Test	Cognitive Domain Assessed	Time to Complete	Screenshot of Task
Variable Difficulty List Memory Test (VLMT)	Recognition Memory	30 s for list presentation	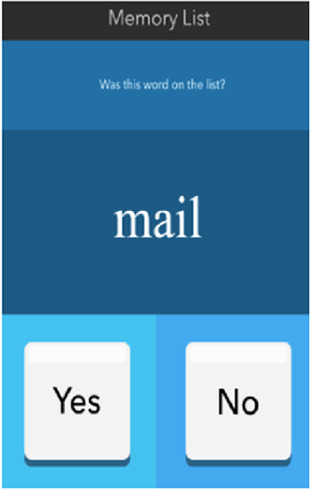
Memory Matrix	Visual Working Memory	Variable; 3 trials; approximately 1–2 min (Mean completion time: 1.5 min	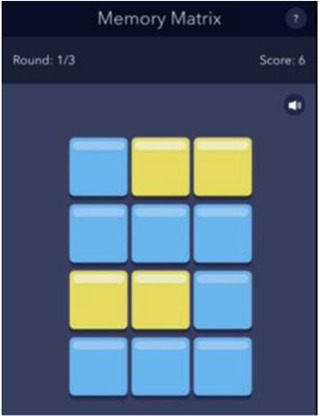
Color-Trick: Meaning-to-Meaning	Executive Function	Variable; 3 trials; approximately 1.5–3 min (Mean completion time: 2.25 min)	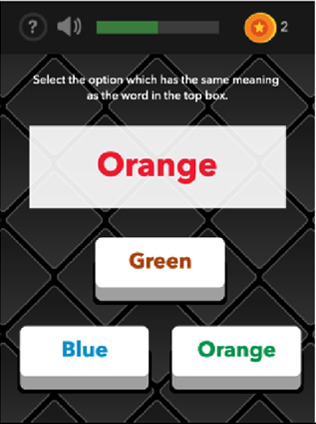
Color-Trick: Meaning-to-Color	Executive Function	Variable; 3 trials; approximately 2–3.5 min (Mean completion time: 2.75 min)	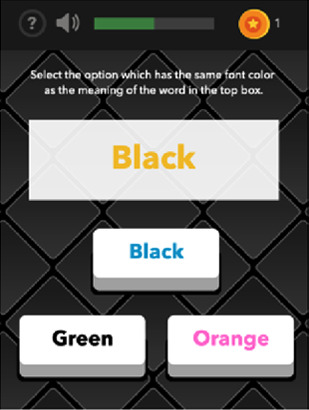
Color-Trick: Yes-No Mechanic	Executive Function	Variable; 3 trials; approximately 2.5–3.5 min (Mean completion time: 3 min)	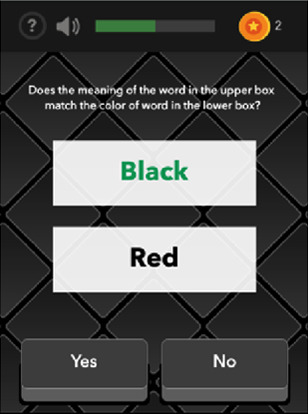

##### 2.3.3.1 Mobile variable difficulty list memory test (VLMT)

The VLMT has been described and validated by Parrish et al. (2020). For this task, participants are presented with a list of words (list length varies between 6, 12, or 18) on 3 separate trials for 30 s each. Immediately following each trial, participants are shown target and distractor words one-by-one and asked to identify whether the word appeared on the list (matched number of target and distractor words presented). Each trial is scored by number of words correctly recalled or based on a percentage of correct target items (range 0%–100%).

##### 2.3.3.2 Memory matrix

During the Memory Matrix task, participants are presented with a matrix of blue tiles. A pattern of yellow tiles is then displayed, and the participant is asked to memorize the location of the yellow tiles. After 1.5 s, the yellow tiles are then switched back to blue, and the participant is asked to tap the tiles that were previously yellow. Matrix sizes are varied across administration days so that participants complete 5 days of 6-tile matrices, 5 days of 12-tile matrices, and 5 days of 18-tile matrices. Each administration also includes three trials of 9 patterns each. Participants earn 1 point for each pattern correctly recreated for a score range of 0–9 per trail and 0–27 per administration.

##### 2.3.3.3 Color trick

The Color Trick task was modelled after the Stroop-type paradigm (Stroop, 1935). Participants completed three different conditions of this task (Meaning-to-Meaning, Meaning-to-Color, Yes-No Mechanic) divided across the 15 days of EMCTs such that each condition was administered 5 times. Each condition includes three trials of 9 items/questions for a total of 27 items per administration. Each item in each condition shows a word in an upper box of the smartphone screen and between 1 and 3 words on the lower half of the screen. The font colors and actual meanings of the upper and lower words are either the same or different colors. The first condition type is *Meaning-to-Meaning*, in which participants are presented with one word in an upper box on their screen and 2–3 word choices on the lower half of their screen and asked to select the word choice that has the same meaning as the word in the top box (e.g., matching top word “pink” with bottom word “pink”). The second condition type is *Meaning-to-Color*, in which participants are presented with one word in an upper box on their screen and 2–3 word choices on the lower box of their screen and asked to select the word choice that has the same font color as the meaning of the word in the top box (e.g., matching top word “pink” with bottom word printed in pink font). The third condition type is *Yes-No Mechanic*, in which participants are presented with one word in an upper box on their screen and one word in a lower box on their screen, and asked, “Does the meaning of the word in the upper box match the color of the word in the lower box?” and the participant can choose either “yes” or “no.” Each trial is scored based on the number of items correct (range 0–9) and average response time for correct items.

### 2.4 Statistical analyses

Demographic differences between groups (MCI+ vs. NCs) and administration formats (in-person vs. remote) were assessed using independent samples *t*-tests or Chi-Square tests (*x*^2^) as appropriate. Adherence was calculated as the percentage of EMA surveys completed by the total number possible (90), as well as the percentage of each of the three mobile cognitive tests completed by the total number possible (15 each). Adherence differences between groups and administration formats were assessed using independent samples *t*-tests. In addition, Pearson's *r* correlations were used to estimate relationships between adherence and demographic differences.

To further assess whether adherence changed over time, we computed missing data variables for the EMCTs that denoted whether participants skipped a test that they were scheduled to take (0 = completed test, 1 = missed test). We then estimated fatigue effects for each of the EMCTs (i.e., whether participants' odds of missing a test was greater on later versus earlier study days) using growth-curve models specified with multilevel logistic regression model in Mplus v. 8.4 (28). Using maximum likelihood estimation, each model regressed participants' log odds of missing a test on time (scaled such that 0 is the midpoint of the EMA period and a one-unit change corresponds to the total change in the log odds of missing a test across the EMA period), MCI status (effect coded such that −1 = NC and 1 = MCI), and the interaction of time with MCI status. Each model also included an unstructured variance-covariance matrix for the random intercepts and slopes. These specifications enabled us to estimate the average probability of missing a test across the EMA period (*via* the threshold value^1^), the average fatigue effect in the sample (*via* the first-order effect of time^2^), whether the average log odds of missing a test across the EMA period differs between NC and MCI (*via* the first-order effect of MCI status), and whether fatigue effects differ between NC and MCI (*via* the interaction of time and MCI status).

We next investigated participants' average performance on the EMCTs across the EMA period. To evaluate group differences (i.e., NC vs. MCI) on EMCT performance across trials, we conducted independent samples t-tests.

The final sets of analyses provided additional psychometric evidence for each EMCT – namely, reliability, convergent validity, ceiling effects, and practice effects. We first calculated Intraclass Correlation Coefficients (ICCs) for each EMCT to quantify the proportion of variance in the tests attributed to trait vs. state components across the EMA period. We then used generalizability theory (see Ref. 29) to estimate the reliability of stable between-person individual differences (*R*_KF_) as well as the reliability of within-person change (*R*_C_) in the EMCT measures that contained multiple trials (i.e., list-learning and matrix memory). These analyses used the Minimum Norm Quadratic Unbiased Estimate (MINQ) method within SPSS v. 26 to estimate the variance components linked to the factorial combination of participant, day, and item (where only participant was treated as a random factor).

We then evaluated the convergent validity evidence for each EMCT by estimating correlations between participants' average performance on a given EMCT and their parallel performance on a similar lab-based measure. Ceiling effects for each EMCT were subsequently evaluated by counting the number of participants who earned the maximum score consistently across the EMA period. Practice effects for each of the EMCTs (i.e., whether participants' performance on the measures systematically changed across the course of the EMA period) were then assessed *via* growth-curve models specified with linear multilevel regression in Mplus v. 8.4 (28). Using maximum likelihood estimation with robust standard errors, each model regressed participants' test scores on time, MCI status, and the interaction of time with MCI status (we used the same scaling for time and MCI status as our analyses investigating fatigue effects). When sufficient variability was present, we specified an unstructured variance-covariance matrix for the random intercepts and slopes. These specifications enabled us to estimate participants' average performance on the EMCT (*via* the intercept), the average practice effect in the sample (*via* the first-order effect of time), whether average levels of performance for an EMCT differs between NC and MCI (*via* the first-order effect of MCI status), and whether practice effects differ between NC and MCI (*via* the interaction of time and MCI status).

## 3 Results

### 3.1 Sample characteristics

Demographic and clinical characteristics by MCI status are displayed in Table 2. Groups were comparable on demographics and did not significantly differ on age, sex, race, ethnicity, or years of education. Groups were also comparable on type of phone used, with 55% of MCI participants and 62% of NCs using iPhones, while the other participants used Android devices (Chi-Square = 11.3, *p *= 0.334; **Supplementary Table S1**).

**Table 2:** Demographics and clinical characteristics by mild cognitive impairment (MCI) status.

	MCI (*n* = 48)	Cognitively Normal (CN) (*n* = 46)	Test-statistic^a^	*p*-value
**Demographics**				
Age in years, *M* (SD); range	72 (7.7); 54–85	70 (6.6); 60–87	0.96	0.34
Sex (% F)	27 (56%)	34 (73%)	3.22	0.07
Race (%)				
White	45 (94%)	41 (89%)	4.81	0.09
Black/African American	1 (2%)	5 (11%)
More than one race	2 (4%)	0 (0%)
Ethnicity (% Hispanic/Latino)	8 (17%)	5 (11%)	0.66	0.42
Education (years), *M* (SD)	16.1 (2.5)	16.2 (2.1)	0.26	0.80
Premorbid IQ (WRAT-4 SS), *M* (SD)	110.2 (15.1)	109.9 (12.0)	0.11	0.91
Employment status				
Retired	26 (54%)	32 (70%)	2.64	0.45
Umemployed	2 (4%)	1 (2%)
Part-time employment or volunteer	14 (29%)	8 (17%)
Full-time employment or volunteer	6 (13%)	5 (11%)
Residential Status				
Independent/Financially Responsible	48 (100%)	44 (96%)	2.13	0.14
Independent/Not Financially Responsible	0 (0%)	2 (4%)
Smartphone used for study				
Personal iPhone	27 (56%)	31 (67%)	4.36	0.11
Personal Android	17 (36%)	15 (33%)
Study Loaned Android	4 (8%)	0 (0%)
Remote Participation	32 (67%)	34 (74%)	0.59	0.44
**Lab-Based Neuropsychological Scores** ^b^				
Hopkins Verbal Learning Test (HVLT) – Immediate Recall	40.7 (9.9)	51.4 (10.0)	5.24	<0.001
Brief Visuospatial Memory Test-R (BVMT-R) – Immediate Recall	50.8 (9.7)
Letter Number Span	45.1 (8.9)	49.6 (9.3)	2.4	0.02
D-KEFS Interference	54.7 (11.4)	56.4 (10.0)	0.73	0.47
**Mobile Cognitive Tests – Mean aggregated scores** ^c^
VLMT 6 words (% Correct)	94.5 (5.7)	95.6 (5.0)	1.04	0.30
VLMT 12 words (% Correct)	85.0 (8.5)	87.3 (6.1)	1.50	0.14
VLMT 18 words (% Correct)	76.6 (9.3)	80.8 (6.9)	2.41	0.02
Memory Matrix (Total Score)	7.3 (0.93)	7.4 (0.83)	0.97	0.33
Color Trick: Meaning-to-Meaning (Total Score)	8.2 (0.51)	8.5 (0.46)	2.13	0.04
Color Trick: Meaning-to-Color (Total Score)	8.6 (0.41)	8.7 (0.42)	1.50	0.07
Color Trick: Yes-No Mechanic (Total Score)	8.6 (0.41)	8.7 (0.28)	1.19	0.24

Note. Values are presented as mean (SD) or *n* (%).

^a^ *T*-tests for continuous variables; Chi square for dichotomous variables.

^b^ Demographically-adjusted *T*-Scores from lab-based neuropsychological scores are reported.

^c^ Raw scores are reported.

Sixty-six participants completed the lab-based neuropsychological visit remotely *via* telehealth, while 28 completed this visit in-person. There were no demographic differences for participants who completed this visit remotely versus in-person except for fewer Hispanic individuals in the in-person group (*χ*^2^ = 6.4, *p *= 0.01). Additionally, there were no significant differences in MCI status (*χ*^2^ = 0.59, *p *= 0.44) or performance on any of the neuropsychological tests based on remote vs. in-person participation (all *ps* > 0.09).

### 3.2 Adherence

For the whole sample, adherence to EMA surveys was 86% (SD = 15.8%; range = 24%–100%). In regard to the mobile cognitive tests, adherence to the VLMT was 84% (SD = 19.3%; range = 7%–100%), adherence to Memory Matrix was 85% (SD = 18%; range = 20%–100%), and adherence to Color Trick was 85% (SD = 17%; range = 13%–100%). Adherence to EMA surveys did not differ by diagnostic status, *t* = 1.21, *p* = 0.23, and neither did completion rates of the mobile cognitive tests (VLMT: *t* = 0.83, *p* = 0.41; Memory Matrix: *t* = 1.56, *p* = 0.12; Color Trick: *t* = 0.97, *p* = 0.33). Further, there was no difference in EMA adherence or mobile cognitive test completion rates for participants who completed the lab-visit remotely or in-person (all *ps* > 0.19). Age, education, and estimated IQ (measured by the WRAT-4) did not correlate with adherence to EMCTs nor with percentage of surveys completed at home or alone, except for a small negative correlation between years of education and completion of the Memory Matrix test. Higher adherence was positively correlated with answering more surveys when home and when alone (see Table 3).

**Table 3:** Correlations between adherence and demographic characteristics in the whole sample (*N* = 94).

	Age	Education	Estimated IQ	% surveys completed at home	% surveys completed alone
EMA Adherence	−0.122	−0.167	−0.129	0.582**	0.286**
VLMT Adherence	−0.029	−0.023	−0.075	0.536**	0.249*
Memory Matrix Adherence	−0.158	−0.205*	−0.129	0.511**	0.274**
Color Trick Adherence	−0.117	−0.114	−0.132	0.363**	0.381**

Note. *p < 0.05; **p < 0.01.

### 3.3 Fatigue effects

Because we used varying list lengths for the VLMT, we included list length (*via* two effect-codes that treated the 18-word list length as the reference group) and its interaction with time as covariates in the VLMT fatigue effect analyses. On average, participants' probability of missing (i.e., failing to complete) a list-learning item was 0.08 for Trial 1 (threshold = 2.40, SE = 0.23, *p* < 0.001), 0.08 for Trial 2 (threshold = 2.38, SE = 0.22, *p* < 0.001), and 0.09 for Trial 3 (threshold = 2.34, SE = 0.22, *p* < 0.001), where trials refer to trials within the same test (e.g., for the VLMT, there were three trials administered at each session). We found no evidence of a fatigue effect for Trial 1 (logit = 0.46, SE = 0.53, *p *= 0.39; OR = 1.58), Trial 2 (logit = 0.56, SE = 0.53, *p* = 0.29, OR = 1.74), or Trial 3 (logit = 0.52, SE = 0.52, *p* = 0.32, OR = 1.68). Moreover, MCI participants did not significantly differ from controls on their log odds of missing a list-learning item vs. not missing the item for Trials 1, 2, or 3 (all *p's* > 0.12) or their fatigue effects for Trials 1, 2, or 3 (all *p's* > 0.57).

Similar to the VLMT, participants' average probability of missing a Memory Matrix item across the EMA period was 0.08 for Trial 1 (threshold = 2.45, SE = 0.21, *p* < 0.001), 0.08 for Trial 2 (threshold = 2.43, SE = 0.21, *p* < 0.001), and 0.08 for Trial 3 (threshold = 2.42, SE = 0.21, *p* < 0.001). Unlike the VLMT, however, we found evidence of fatigue effects for the Memory Matrix items across the three trials. In particular, participants' odds of missing a Memory Matrix item vs. not missing a Memory Matrix item from the beginning to the end of the EMA period increased approximately 3.23-fold for Trial 1 (logit = 1.174, SE = 0.52, *p* = 0.023), approximately 3.47-fold for Trial 2 (logit = 1.244, SE = 0.51, *p* = 0.014), and approximately 3.42-fold for Trial 3 (logit = 1.231, SE = 0.50, *p* = 0.014). That is, whereas participants' probability of missing a Memory Matrix item was 0.05 at the beginning of the EMA period for Trials 1, 2, and 3, their probability of missing a Memory Matrix item at the end of the EMA period was 0.13 for Trials 1 and 2 and 0.14 for Trial 3. Nonetheless, MCI participants did not significantly differ from controls on their log odds of missing a Memory Matrix item vs. not missing the item for Trials 1, 2, or 3 (all *p's* > 0.06) or on their fatigue effects for Trials 1, 2, or 3 (all *p's* > 0.59).

Participants’ average probability of missing a Color Trick item across the EMA period was 0.09 for Trial 1 (threshold = 2.285, SE = 0.19, *p* < 0.001), 0.09 for Trial 2 (threshold = 2.269, SE = 0.19, *p* < 0.001), and 0.09 for Trial 3 (threshold = 2.256, SE = 0.19, *p* < 0.001). We found no evidence of a fatigue effect for Trial 1 (logit = 0.299, SE = 0.46, *p* = 0.514, OR = 1.35), Trial 2 (logit = 0.242, SE = 0.46, *p* = 0.598, OR = 1.27), or Trial 3 (logit = 0.269, SE = 0.45, *p* = 0.55, OR = 1.31). MCI participants also did not significantly differ from controls on their log odds of missing a Color Trick item vs. not missing the item for Trials 1 to 3 (all *p*'s > 0.07) or on their fatigue effects for Trials 1 to 3 (all *p*'s > 0.20).

### 3.4 EMCT performance and group differences

Table 2 presents average mobile cognitive test performance for the MCI and NC groups across the EMA period. As expected, participants generally committed more errors on the VLMTs when the list length was greater. Participants' performance on the Memory Matrix and Color Trick tests was also quite high. While participants with MCI scored lower on all EMCTs, they only performed significantly worse than the NC participants on the 18-word VLMT and the Color Trick: Meaning-to-Meaning task.

We also examined performance differences by phone type. In the overall sample, there were no significant performance differences based on phone type (**Supplementary Table S2**). When examining the effects of both phone type and group (and their interaction) on mobile cognitive test performance, no main effects were found for the VLMT 6- or 12-word list, Memory Matrix, Color Trick Meaning-to-Color, or Color Trick Yes-No Mechanic (all *p's* > 0.05). Further, there were no significant interactions between phone type and group on any of the mobile cognitive tests (all *p's* > 0.05). For the VLMT 18-word list, a main effect for group was observed, such that NC participants performed better than participants with MCI (F = 6.53, *p *= 0.01); there was no main effect for phone type (*F* = 0.53, *p *= 0.47). Lastly, there was a main effect for group on Color Trick Meaning-to-Meaning, such that MCI participants performed worse than NC participants (*F* = 5.23; *p *= 0.03), but there was no main effect for phone type (*F* = 0.11, *p *= 0.74).

### 3.5 EMCT psychometrics: Reliability, convergent validity, ceiling effects, and practice effects

#### 3.5.1 Psychometric evidence for VLMT

Aggregated across trials, the Intraclass Correlation Coefficients (ICCs) for each trial length of the VLMT were 0.22, 95% CI [0.11, 0.32] for the 6-word list, 0.33, 95% CI [0.22, 0.44] for the 12-word list, and 0.32, 95% CI [0.20, 0.42] for the 18-word list. Thus, most of the variance on VLMT can be attributed to within-person differences in performance across trials. Using generalizability theory, we further found that the reliability of stable between-person individual differences in VLMT scores across list lengths and trials was quite high (*R*_KF_ = 0.94). In contrast, the reliability of within-person change across list lengths and trials was somewhat low (*R*_C_ = 0.57).

To examine convergent validity, we examined relationships between the VLMT with immediate recall scores from the HVLT and BVMT (see Table 4). We examined the VLMT data in two ways: percentage correct by trial length and overall correct across all trial lengths. In the overall sample, percent of items correct on the 18-item VLMT list was positively correlated with the HVLT (*r* = 0.33, *p *< 0.001). The relationships between the 6- and 12-item percent correct VLMT lists were not significantly related to HVLT performance. When looking at the overall correct data across all three list lengths, the VLMT was positively associated with HVLT (*r* = 0.26, *p* = 0.012). When comparing the VLMT to the BVMT, percent of items correct on the 6-item VLMT list was positively correlated with the BVMT (*r* = 0.27, *p* = 0.01); 12- and 18-item VLMT lists were unrelated to the BVMT. The VLMT overall correct scores (across all three list lengths) was positively correlated with BVMT performance (*r* = 0.27, *p* = 0.01).

**Table 4:** Correlations between mobile cognitive tests and in-lab neuropsychological performance in whole sample (*N* = 94).

Mobile Cognitive Tests (Raw Scores)	Demographic Characteristics				Lab Administered Neuropsychological Tests				
	Age	Sex	Race	Education	WRAT-4	HVLT-Immediate Recall	BVMT-Immediate Recall	Letter Number Span	D-KEFS Color-Word Interference Test (time)
VLMT 6 words (% Correct)	−0.27*	0.25*	0.11	0.04	0.07	0.12	0.27**	0.23*	−0.29*
VLMT 12 words (% Correct)	−0.17	0.09	0.11	0.04	−0.03	0.13	0.09	0.07	−0.17
VLMT 18 words (% Correct)	−0.12	0.24*	0.02	0.04	−0.04	0.33**	0.17	0.03	−0.020
VLMT Overall Mean (all trials)	−0.01	0.37**	0.01	0.08	0.17	0.26**	0.27**	0.10	−0.29*
Memory Matrix (Total Score)	−0.43**	0.09	0.11	0.21*	0.04	0.20	0.17	0.38**	−0.26*
Color Trick: Meaning-to-Meaning (Total Score)	−0.12	0.24*	0.13	0.28**	0.30**	0.28**	0.32**	0.24*	−0.33**
Color Trick: Meaning-to-Color (Total Score)	−0.05	0.18	0.03	−0.25*	0.22*	0.21*	0.29**	0.18	−0.19
Color Trick: Yes-No Mechanic (Total Score)	−0.04	0.23*	0.07	0.33**	0.28**	0.21*	0.19	0.20	−0.18

Note. *p < 0.05; **p < 0.01.

We next examined whether there were ceiling effects at any of the VLMT list lengths. At length 6, there was some evidence for ceiling effects such that on Trial 1, 13 (28%) NC and 15 (31%) MCI participants consistently scored 100%; on Trial 2, 23 (50%) NC and 26 (54%) MCI consistently scored 100%; and on Trial 3, 29 (63%) NC and 27 (56%) MCI participants consistently scored 100%. No ceiling effects were observed for list length 12 or 18.

Practice effects were subsequently investigated with linear mixed effect models to determine whether participants' performance on the VLMT systematically changed across the EMA period.^3^ Note that all effects were adjusted for list length. On average, participants recognized 10.06 out of an average of 12 words (i.e., average of 6, 12, and 18) correctly (SE = 0.08), averaging across the list lengths. Moreover, participants showed a systematic decline in the number of words they got correct for the list-learning task across the EMA period (on average, participants' total change = −0.84, SE = 0.14, *p* < 0.001). Although MCI participants (*M* = 9.87) significantly differed from controls (*M* = 10.25) on their average number of words correct across the trials (*b* = −0.19, SE = 0.08, *p* = 0.015), participants' systematic change in words correct across the EMA period was not significantly related to MCI status (*b* = −0.10, SE = 0.14, *p* = 0.471).

#### 3.5.2 Psychometric evidence for the Memory Matrix task

The ICC for the average Memory Matrix score across trials was 0.07, 95% CI [0.03, 0.11], indicating that the majority of the variance on this measure can be attributed to within-person differences in performance across trials. Generalizability theory analyses further showed that the reliability of stable between-person individual differences was 0.97. The reliability of within-person change was also satisfactory, with a value of 0.72.

To assess convergent validity, we looked at associations between the Letter-Number Span and performance on Memory Matrix. Memory Matrix scores were positively and significantly correlated with Letter-Number Span (*r* = 0.38, *p* < 0.001). Relationships with demographics and the other lab-administered tests are presented in Table 3.

Although we did not find any evidence of a ceiling effect for Memory Matrix, we nonetheless decided to modify our analyses for the practice effects to account for the possibility of right-hand censoring in the data. Because participants' average scores on these EMCTs tended to be close to the maximum number correct, we wanted to ensure that the growth-curve analyses could accurately capture systematic changes in performance across the EMA period in spite of any measurement limitations. As such, these analyses use Mplus v. 8.4 to estimate what the scores would be if there was not an upper limit (e.g., scores can be greater than 9).

Averaging across trials, participants were estimated to get 8.43 items correct on average out of 10 (SE = 0.12, *p* < 0.001). Moreover, participants showed systematic change in the number of Memory Matrix items they got correct across the EMA period (on average, participants' total change = 1.75, SE = 0.20, *p* < 0.001). However, MCI participants did not significantly differ from NCs on either the intercepts (*b* = −0.16, SE = 0.13, *p* = 0.22) or the slopes (*b* = −0.11, SE = 0.20, *p* = 0.577). In addition, although the data suggest evidence of a practice effect, closer inspection of participants' trajectories *via* spaghetti plots suggests that participants' performance on the Memory Matrix ebbs and flows throughout the EMA period. Specifically, there appears to be a slight decrease in performance from days 1 to 13, then a marked improvement in performance from days 13 to 21, and then a slight decrease in performance from days 21 to 30.

#### 3.5.3 Psychometric evidence for the Color Trick task

We computed ICCs for participants' accuracy on each version of the Color Trick task: Meaning-to-Meaning, ICC = 0.13, 95% CI [0.07, 0.18]); Meaning-to-Color, ICC = 0.17, 95% CI [0.10, 0.23]; and Yes-No Mechanic, ICC = 0.23, 95% CI [0.15, 0.30]), indicating that the majority of the variance on these measures can be attributed to within-person differences in performance across trials. Table 3 presents associations between the Color Trick tasks with demographics and lab-based assessments. As can be seen, the D-KEFS Interference Trial showed a moderate negative correlation with the Meaning-to-Meaning Color Trick task, such that faster performance on the D-KEFS was related to better performance on Meaning-to-Meaning.

We next examined whether there were ceiling effects for participants' accuracy on any of the Color Trick tasks. There was some evidence for ceiling effects, such that 5 (11%) NC and 1 (2%) MCI participants consistently scored 100% for the Meaning-to-Meaning task; 8 (17%) NC and 4 (8%) MCI participants consistently scored 100% for the Meaning-to-Color task; and 5 (11%) NC and 5 (10%) MCI participants consistently scored 100% for the Yes-No Mechanic task. To account for the possibility of right-hand censoring in the data, we adapted our practice effect analyses for the color trick tasks to be consistent with the modifications we made for the memory matrix task analyses.

For Meaning-to-Meaning trials, participants were estimated to get 9.86 items correct on average (SE = 0.16, *p* < 0.001). Moreover, participants showed systematic change in the number of items they got correct across the EMA period (on average, participants' total change = 2.19, SE = 0.32, *p* < 0.001). Although MCI participants (*M* = 9.51) significantly differed from NCs (*M* = 10.21) on their average number of items correct across the EMA period (*b* = −0.35, SE = 0.14, *p* = 0.011), participants' systematic change in the number of items that they got correct across the EMA period was not significantly related to MCI status (*b* = −0.12, SE = 0.30, *p* = 0.677).

For Meaning-to-Color trials, participants were estimated to get 11.01 items correct on average (SE = 0.23, *p* < 0.001). Moreover, participants showed systematic change in the number of items they got correct across the EMA period (on average, participants' total change = 1.75, SE = 0.42, *p* < 0.001). Similar to performance on Meaning-to-Meaning trials, MCI participants (*M* = 10.62) significantly differed from NCs (*M* = 11.40) on their average number of items correct across the EMA period (*b* = −0.39, SE = 0.16, *p* = 0.015). In addition, participants' systematic change in the number of items correct across the EMA period was not significantly related to MCI status (*b* = 0.53, SE = 0.37, *p* = 0.154).

Lastly, for Yes-No Mechanic trials, participants were estimated to get 11.05 items correct on average (SE = 0.21, *p* < 0.001). Participants also showed systematic change in the number of items they got correct across the EMA period (on average, participants' total change = 0.91, SE = 0.43, *p* = 0.035). MCI participants did not significantly differ from NC on either the intercepts (*b* = −0.29, SE = 0.15, *p* = 0.06) or the slopes (*b* = −0.04, SE = 0.36, *p* = 0.902).

## 4 Discussion

This study evaluated the feasibility and validity of three mobile cognitive tests among persons with and without MCI. Adherence to this 30-day, fully remote, ecological momentary cognitive testing protocol was very good, with 86% of assigned EMA sessions completed and 84–85% of mobile cognitive testing sessions completed. In this sample of cognitively normal and cognitively impaired older adults, adherence did not differ by MCI status. Further, these findings indicate adherence does not differ by demographic characteristics. Participants who had higher adherence answered more surveys when home and alone compared to people with lower adherence.

We found mixed findings of a fatigue effect at the level of the individual tests, such that there was no evidence of a fatigue effect for the VLMT or Color Trick tests, but participants were more likely to miss Memory Matrix tests over the course of the 30-day protocol (with no difference by NC vs MCI). In another study using the VLMT and Memory Matrix test (14-day protocol in participants with bipolar disorder and control participants) we found an overall fatigue effect for the EMCT protocol, such that participants were more likely to miss a test as study day increased (no differences by diagnostic status), but we did not examine fatigue effects at the level of the individual test (30). Of note, the prior study had a more intensive protocol than the current study, with participants pinged to complete 2–3 mobile cognitive tests three times daily for 14-days. When designing EMCT protocols there is always a frequency and duration trade-off when considering participant burden and capturing outcomes of interest. Our prior work has shown that a 14-day period is sufficient to capture cognition and mood data across various contexts (e.g., 31–35), and other groups have demonstrated strong feasibility and psychometric properties for measuring cognition in as few as 7–8 days (e.g., 14, 16). In general, the 30-day EMCT protocol in this study was largely well tolerated and provides further support for the feasibility of remote, smartphone-based cognitive testing among older adults. Participants had higher rates of adherence than has been reported with other digital health apps (36), which is likely due to a combination of factors including incentives for completing each testing session, brief, gamified tests that varied in difficulty, establishment of good rapport with the study team, and a time-limited engagement with the app.

The psychometric properties of the tasks in this sample were generally good. The reliability of stable between-person individual differences for the VLMT and Memory Matrix were very high, indicating that participants' averaged scores on each mobile cognitive test across the EMA period can reliably assess differences between participants' average levels of the variables. In addition, although the reliability of within-person change (i.e., the consistency in the degree of systematic within-person change across multiple items over time) for Memory Matrix was adequate, the corresponding reliability estimate for the VLMT was not. Of note, the reliability of within-person change would likely increase if there were more trials, but this would also increase participant burden. As hypothesized, the VLMT overall percentage correct score had an overall moderate positive correlation with the HVLT and BVMT, demonstrating convergent validity. Further, MCI participants recognized significantly fewer words on this task than CN participants. The trajectories of word recognition did not differ by group status across the 30-day study period, but rather, on average, the participants with MCI remembered fewer words overall. In the whole sample, females performed significantly better than males on both the VLMT and HVLT, which is consistent with the female verbal memory advantage highlighted in the Alzheimer's disease literature (e.g., 37), and further supports utility of the VLMT in people with MCI.

Also consistent with our hypotheses, Memory Matrix had a moderately positive correlation with Letter Number Span. Group differences in Memory Matrix performance were not found, although the data did demonstrate variability in performance on this task over the 30-day study period, and future work is needed to examine whether context (e.g., home vs. away from home; alone vs. with others; time of day effects) affected performance on this task. Lastly, data from the Meaning-to-Meaning condition of the Color Trick task was related to faster performance on the D-KEFS Interference Trial. The other two Color Trick conditions were not significantly related to D-KEFS performance. For the Meaning-to-Meaning and Meaning-to-Color trials, MCI participants performed significantly worse than NCs. There was some evidence for ceiling effects, especially among the NC participants, for all versions for Color Trick, and future development of this task, such as increasing the number of trials at each administration or increasing difficulty of the task, may be beneficial if this task is to be adopted in a cognitively normal sample. It is worth noting that traditional neuropsychological tests, albeit used as the “gold standard” comparison for mobile cognitive tests in this study, are limited in that they only provide a snapshot of cognitive abilities at one time point. We would not expect a high correlation between once-administered tests and averaged mobile cognitive testing performance. Additional research is needed to examine whether one testing method is superior to the other when examining clinical outcomes such as disease progression, medication effects, reversion rates, and associations with pathology.

This study is not without limitations. Our sample was largely White and highly educated, which may limit generalizability. There were significantly more women in the cognitively normal group compared to the MCI group, which could have an effect on our findings, especially given the female advantage to verbal memory. Future work is needed with larger and more representative samples to determine whether these tests would be appropriate to detect differences based on cognitive status in randomized controlled trials. Additionally, data were collected during the COVID-19 pandemic, and we did not measure how pandemic-related factors may have influenced performance on these tasks. Another limitation that applies to all ambulatory mobile cognitive testing is that it is difficult to identify suspected cheating, such as whether the participant or someone else took the tests. Relatedly, it is difficult to assess effort on mobile cognitive tests. However, aggregating mobile cognitive test scores can reduce error associated with instances of low effort, as evidenced by the construct validity findings of our mobile cognitive tests with lab-based tests. We did observe evidence of ceiling effects on the VLMT 6-item list and the Color-Trick task in the whole sample, and these trials could possibly be adapted to be made more difficult or used as performance-validity tests in future EMCT protocols. A final limitation is that while we were able to examine differences by smartphone make (iOS vs. Android), we did not have a sufficient sample size to examine differences by smartphone model or OS version, service providers, connectivity, and screen size, all of which may impact response times. Touch sensitivity and latency can differ by up to 100 ms between difference devices, especially between newer and older devices (38, 39). In this study none of the mobile cognitive test outcomes were based on speed. In future work examining timing of responses, these smartphone differences should be examined.

In conclusion, our data add to the extant literature on self-administered mobile cognitive testing in older adults, and is one of the first studies examining an EMCT protocol in people with MCI. The tests are automatically scored, integrated with EMA surveys, and available on iOS and Android operating systems for ease of use by other investigators. Adherence to the EMCTs was high, and the psychometric data are promising. Thus, the three mobile cognitive tests in this study, and particularly the VLMT, may serve as useful tools in future clinical trials with cognition as an endpoint, especially in persons with increased risk for Alzheimer's disease such as those with MCI.

## Data Availability

The original contributions presented in the study are included in the article/**Supplementary Files**, further inquiries can be directed to the corresponding author/s.

## Footnotes

^1^Participants’ average probability of missing an EMCT item was computed as 1/(1 + exp(*τ*)), where *τ* = threshold.

^2^In cases where there was evidence for a fatigue effect, we used the following formula to determine participants’ average probability of missing an EMCT item at the beginning (time = −0.50) and end (time = 0.50) of the study:

$$\pi =\frac{\exp\left[-\left(\tau \right)+\;\beta_{1}X_{i}\right]}{1+\exp\left[-\left(\tau \right)+\;\beta_{1}X_{i}\right]}$$

where τ = threshold, β1 = Slope reflecting fatigue effect, and Xi = the specific value of time.

^3^Practice effects were treated as fixed effects as opposed to random given limited variability in the data set.
