# Supplementary material for: Feasibility and validity of ecological momentary cognitive testing among older adults with mild cognitive impairment
Source: Front Digit Health. 2022 Aug 5;4:946685. doi: 10.3389/fdgth.2022.946685 (PMC9390883; doi:10.3389/fdgth.2022.946685)
Supplement: Supplementary file 1 [file Tables.docx]

Supplemental Table 1. Phone Type and Operating System by Group.

| Phone Type and Operating System (OS) version | Mild Cognitive Impairment (MCI) | Normal Cognition (NC) |
| --- | --- | --- |
| Android OS 6 | 1 | 0 |
| Android OS 7 | 0 | 2 |
| Android OS 8 | 2 | 2 |
| Android OS 9 | 8 | 2 |
| Android OS 10 | 4 | 5 |
| Android OS 11 | 3 | 4 |
| iOS 10 | 0 | 1 |
| iOS 12 | 0 | 1 |
| iOS 13 | 1 | 0 |
| iOS 14 | 20 | 27 |
| iOS 15 | 2 | 1 |

*Note.* Data were missing for 1 NC and 7 MCI participants.

Supplemental Table 2. Mean Performance on the Mobile Cognitive Tests by Phone Type

| Mobile Cognitive Tests, M(SD); range | Phone Type | | F(df)^a^ | *P*-value |
| --- | --- | --- | --- | --- |
|  | Android (N=36) | iPhone  (N=58) |  |  |
| VLMT 6 words, % correct | 0.94(0.06);  0.81-1.00^a^ | 0.96(0.05);  0.76-1.00^c^ | 2.16(90) | 0.14 |
| VLMT 12 words, % correct | 0.86(0.08);  0.62-0.97 | 0.86(0.07);  0.60-0.97 | .13(92) | 0.72 |
| VLMT 18 words, % correct | 0.79(0.08);  0.62-0.94^a^ | 0.78(0.09);  0.54-0.91 | .11(91) | 0.74 |
| Memory Matrix Total score | 7.20(0.99);  4.21-8.41 | 7.41(0.81);  5.44-8.74 | 1.27(92) | 0.26 |
| Color Trick: Meaning-to-Meaning Total score | 8.34(0.59);  6.42-9.00 | 8.34(0.43);  7.27-9.00 | .001(92) | 0.98 |
| Color Trick: Meaning-to-Color Total score | 8.52(0.53);  6.83-9.00 | 8.68(0.33);  7.73-9.00 | 3.26(92) | 0.07 |
| Color Trick: Yes-No Mechanic Total score | 8.63(0.53);  6.17-9.00 | 8.72(0.28);  7.86-9.00 | 1.25(92) | 0.27 |

*Note.*

VLMT = Variable Difficulty List Memory Test

^a^N=31

^b^N=57
